# Macrophage inducible nitric oxide synthase circulates inflammation and promotes lung carcinogenesis

**DOI:** 10.1038/s41420-018-0046-5

**Published:** 2018-03-26

**Authors:** Xin Wang, Zane Gray, Jami Willette-Brown, Feng Zhu, Gongping Shi, Qun Jiang, Na-Young Song, Liang Dong, Yinling Hu

**Affiliations:** 1 Cancer and Inflammation Program, Center for Cancer Research, National Cancer Institute, National Institutes of Health, Frederick, MA 21701 USA; 2 Thoracic and Gastrointestinal Oncology Branch, Center for Cancer Research, National Cancer Institute, National Institutes of Health, Bethesda, MD 20892 USA; 3 Department of Respiratory Medicine, Qilu Hospital of Shandong University, 107#, Wenhua Xi Road, Jinan, 250012 Shandong China; 4 The Respiratory Department, Jinan Central Hospital Affiliated to Shandong University, Jinan, Shandong 250013 China

## Abstract

Human lung squamous cell carcinoma (SCC) is highly associated with increased pulmonary macrophage infiltration. Previously, we showed that marked pulmonary infiltrating macrophages were required for spontaneous lung SCC development in a mouse model (*L-Ikkα*^*KA/KA*^, *KA/KA*) that resembles human lung SCC. Interestingly the lung SCC-associated macrophages specifically express elevated inducible nitric oxide synthase (NOS2). However, the role of macrophage NOS2 in lung carcinogenesis has not been explored. Here, we show that NOS2 ablation inhibits macrophage infiltration, fibrosis, and SCC development in the lungs of *KA/KA* mice. Macrophage NOS2 was found to circulate inflammation and enhance macrophage migration and survival. NOS2 promotes foamy macrophage formation characterized with impaired lipid metabolism. NOS2 null bone marrow transplantation reduces foamy macrophage numbers and carcinogenesis in *KA/KA* chimaeras. This finding sheds light on a new mechanism by which macrophage NOS2 increases pulmonary inflammatory responses and macrophage survival and impairs macrophage lipid metabolism, thereby promoting lung SCC formation.

## Introduction

Lung cancer is a leading cause of all cancer-related deaths worldwide. Lung squamous cell carcinoma (SCC) is one of the predominant types of lung cancer^1^. Epidemiological studies have shown that most patients with lung SCC have a history of cigarette smoking^2^. Recently, two spontaneous lung SCC mouse models demonstrate that a robust chronic inflammatory response is associated with lung SCC^3,4^. Depleting macrophages prevents lung SCC development in *L-Ikkα*^*KA/KA*^ (*KA/KA*) mice^3^, indicating that these macrophages act as a driving force for the pathogenesis of lung SCC. Interestingly, these pathogenic macrophages express marked inducible nitric oxide synthase (iNOS/NOS2); however, the effect of macrophage NOS2 on lung tumorigenesis is largely unclear.

Elevated NOS2 levels have been observed in human lung cancer, as well as breast, brain, prostate, colorectal, and pancreatic carcinomas^5–7^. Nitric oxide (NO) is one of the major resources of oxidative stress. NOS catalyzes l-arginine to produce NO and l-citrulline. There are three isoforms of NOS: neuronal NOS (NOS-1), inducible NOS (NOS2), and endothelial NOS (NOS-3). The expression of NOS-1 and NOS-3 is constitutive and calcium/calmodulin-dependent and they generate NO in the picomolar–nanomolar range. NOS2 in macrophages is induced by TNFα, IL-1 (interleukin-1), IFNγ, endotoxins, or lipopolysaccharide (LPS) stimulation, and locally generates high-output quantities of NO at micromolar range for prolonged periods of time. A NOS2 level may reflect the status of inflammation. The excessive oxidative stress and inflammation thus form a loop, which creates a tumor microenvironment and promotes carcinogenesis.

Cigarette smoke exposure can lead to lipid accumulation in macrophages, which drives pulmonary inflammation^8^. The impaired lipid metabolism results in cytoplasmic needle-shaped crystalline bodies and greatly enlarged macrophage sizes (called foamy macrophages) that express Ym-1 (or chitinase-3-protein 3, Chi3l3), a marker for M2 macrophage^9^. The impaired lipid metabolism has been reported in human and mouse lung SCC^10^. NOS2 expression is minimal in macrophages until these cells are stimulated^11^. A substrate competition for l-arginine exits between the NOS- and arginase 1-mediated pathways because arginase 1 converts l-arginine to l-ornithine and urea, diminishing NO synthesis^9^. The balance of NOS2 and arginase 1 regulates macrophage differentiation^12^. To date, the effect of NOS2 on the lipid metabolism remains to be fully explored. Because increased cytokines can induce NOS2 expression^12^, it still needs to be examined whether the macrophage NOS2 contributes to a lung tumor microenvironment in *KA/KA* mice^3^ or is a response to increased pulmonary inflammation during lung carcinogenesis.

In this study, we show that NOS2 ablation inhibits pulmonary inflammation, lung epithelial cell overgrowth, and lung SCC development associated with markedly decreased pulmonary macrophage/foamy macrophage numbers in *KA/KA* mice. NOS2 null bone narrow (BM) transplantation decreases the pulmonary macrophage numbers and inhibits lung SCC incidence in irradiated *KA/KA* chimeric mice. Also, lung epithelial cell NOS2 is required for maintaining microenvironmental inflammation. Together, these results reveal a crucial role of macrophage NOS2 for circulating inflammatory responses and promoting lung SCC development.

## Results

### NOS2 ablation inhibits inflammatory responses and spontaneous lung SCC in *KA/KA* mice

**Fig. 1:**
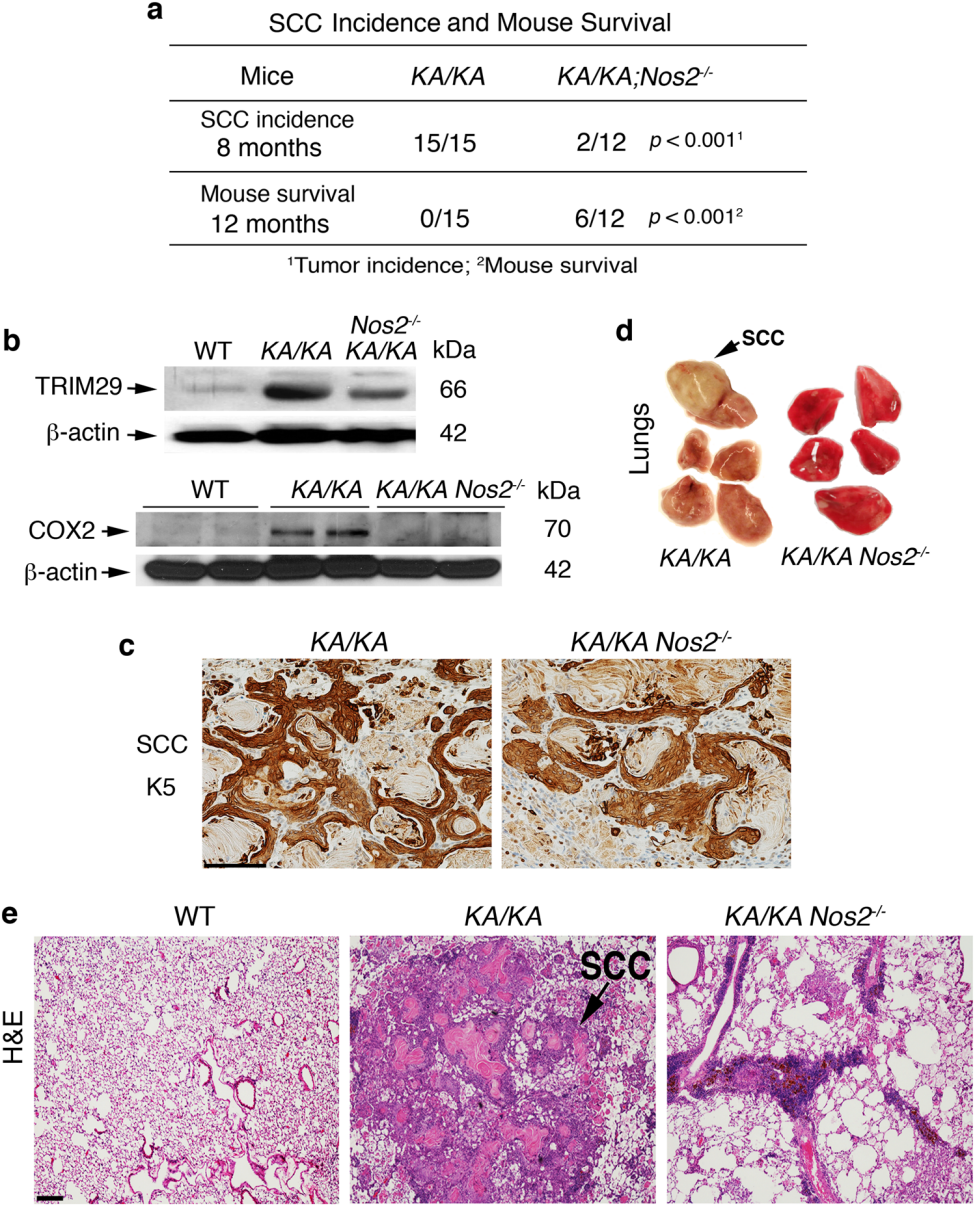
NOS2 deletion reduces lung SCC development. **a** Comparison of lung SCC incidence in *KA/KA* and *KA/KA;Nos2*^*−/−*^ mice at 8 months of age and mouse survival at 12 months of age. *P-*value between *KA/KA* and *KA/KA*;*Nos2*^*−/−*^ groups is analyzed by chi-square test. **b** Western blotting shows TRIM29 and COX2 levels in WT, *KA/KA*, and *KA/KA;Nos2*^*−/−*^ lungs. β-actin, protein loading control. **c** K5 immunohistochemistry (IHC) stains lung SCCs from *KA/KA* and *KA/KA*;*Nos2*^*−/−*^ mice. K5 keratin 5; dark brown, positive staining. Scale bar, 50 μm. **d** The appearances of lungs of *KA/KA* and *KA/KA;Nos2*^*−/−*^ mice. An arrow indicates a lung SCC. **e** Hematoxylin and Eosin (H&E) examination of lungs of WT, *KA/KA*, and *KA/KA;Nos2*^*−/−*^ mice. Scale bar, 40 μm

To evaluate the role of NOS2 in lung SCC development, we crossed *KA/KA* mice with *Nos2*^*−/−*^ mice for more than five generations and generated *KA/KA;Nos2*^*−/−*^ mice on the FVB background. *KA/KA* mice at 4–10 months of age develop spontaneous lung SCCs that express lung SCC markers of keratin 5 (K5) and increased TRIM29 and these mice die before 10 months due to lung SCCs or severe systemic inflammation^3^ (Fig. 1a–c). Whereas, *KA/KA*;* Nos2*^*−/−*^ mice lived longer and developed significantly less lung SCCs than *KA/KA* mice (Fig. 1a). Two out of 12 *KA/KA;Nos2*^*−/−*^ mice developed lung SCCs before 8 months of age and six of 12 *KA/KA;Nos2*^*−/−*^ mice lived to one year old (Fig. 1a). Lung SCCs derived from *KA/KA* and *KA/KA*;*Nos2*^*−/−*^ mice expressed K5 and TRIM29, but NOS2 deletion reduced TRIM29 and COX2 expression in *KA/KA* lungs (Fig. 1b). *KA/KA;Nos2*^*−/−*^ lungs became soft with normal red color compared to *KA/KA* lungs that show yellow color^3,13^ (Fig. 1d).

Furthermore, the weights of *KA/KA* lungs and spleens were significantly increased compared to wild type (WT), but the weights of *KA/KA;Nos2*^*−/−*^ lungs and spleens were slightly reduced compared to *KA/KA* ones (Supplementary Figures S1a and b). We observed increased infiltrating cells in *KA/KA;Nos2*^*−/−*^ lungs compared to WT lungs (Fig. 1e; Supplementary Figure S1c). Although some *KA/KA;Nos2*^*−/−*^ mice did not bear lung SCCs and could survive to 12 months, they still suffered from other phenotypes. Thus, we were not able to maintain these mice. Together, these results suggest that NOS2 deletion decreases lung SCC incidence.

### NOS2 deletion reduces basal epithelial cell hyperproliferation, fibrosis, and inflammatory responses in the lungs of *KA/KA* mice

**Fig. 2:**
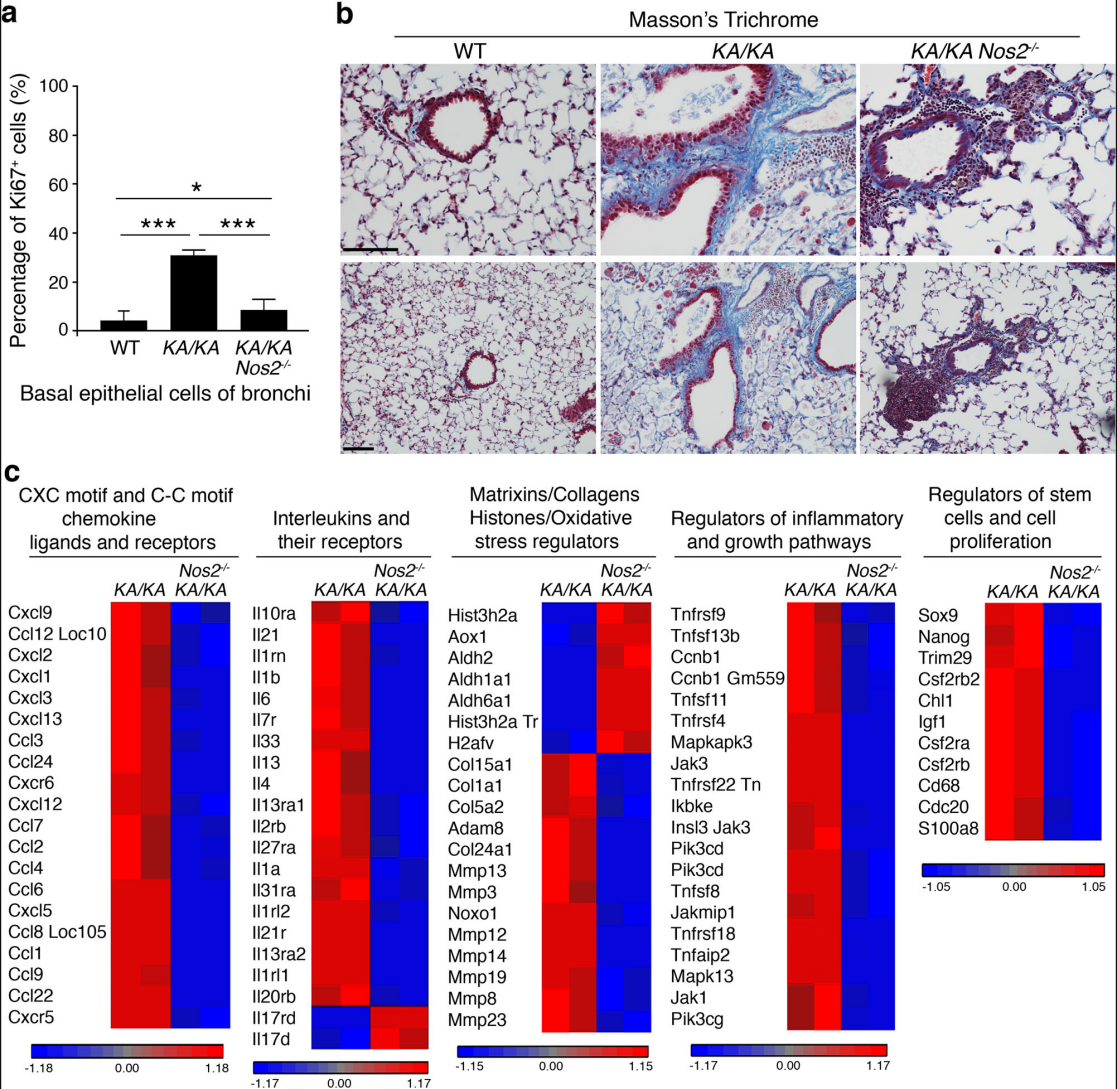
NOS2 deletion inhibits lung epithelial cell hyperproliferation, fibrosis, and inflammatory responses. **a** Analysis of Ki67-positive epithelial cells in basal cells of lung bronchi in WT, *KA/KA*, and *KA/KA;Nos2*^*−/−*^ mice, examined by IHC staining and statistically analyzed by Student’s *t*-test. Data represent mean ± SD (*n* = 3/group, 500 cells were counted in each group). *p < 0.05; ***p < 0.001. **b** Chemical Masson’s trichrome staining in the lungs of WT, *KA/KA*, and *KA/KA;Nos2*^*−/−*^ mice at 5 months of age. Blue color, positive staining. Scale bar, 50 μm. **c** Heat maps show gene expression profiles between *KA/KA* and *KA/KA*;*Nos2*^*−/−*^ lungs from mice at 5 months of age. All the groups between *KA/KA* and *KA/KA*;*Nos2*^*−/−*^ lungs were statistically analyzed by an ANOVA one-way test. Each *p*-value for all groups, *p* < 0.05

Lung SCC is derived from the basal cells of lung bronchi. Ki67-stained proliferative cells in the basal layer of upper airway epithelium were significantly increased in *KA/KA* lungs, compared to WT lungs, and NOS2 deletion decreased Ki67-positive basal epithelial cells in the upper airways of *KA/KA;Nos2*^*−/−*^ lungs compared to *KA/KA* lungs (Fig. 2a and Supplementary Figure 2a). Increased oxidative stress is associated with fibrosis^13^. *KA/KA* lungs showed yellow color and *KA/KA;Nos2*^*−/−*^ lungs showed red color (Fig. 1d), suggesting that induced NOS2 levels in *KA/KA* lungs may contribute to fibrosis development. Masson’s trichrome staining for collagen, a fibrosis marker, showed increased collagen staining in *KA/KA* lungs compared to WT and that NOS2 deletion reduced collagen expression (Fig. 2b), indicating that induced NOS2 contributes to the development of lung fibrosis.

We further analyzed gene expression profiles in *KA/KA* and *KA/KA*;*Nos2*^*−/−*^ lungs (Fig. 2c, accession number: GSE101759). NOS2 deletion-mediated changes include reduced expression of many chemokines (CXC and CC), cytokines (IL), matrix metalloproteinases, stemness cell markers, cell cycle/mitogenic activators, and TNF and Jak inflammatory signaling pathways, but increased molecules that were involved in regulating cellular events, such as cell growth and adhesion (Fig. 2c). These major inflammation-related molecular alterations may contribute to the reduction of lung SCC and fibrosis development although *KA/KA;Nos2*^*−/−*^ mice still remain other phenotypes. In addition, numbers of T cells and B cells were comparable in the lungs of *KA/KA* and *KA/KA;Nos2*^*−/−*^ mice (Supplementary Figures S2b and c). Previously, we demonstrated that macrophage infiltration is required for lung SCC development in KA/KA mice^3^. Thus, we focused on understanding the effect of macrophage NOS2 on lung SCC development.

### NOS2 deletion reduces numbers of macrophages and foamy macrophage with impaired lipid metabolism in the lungs

**Fig. 3:**
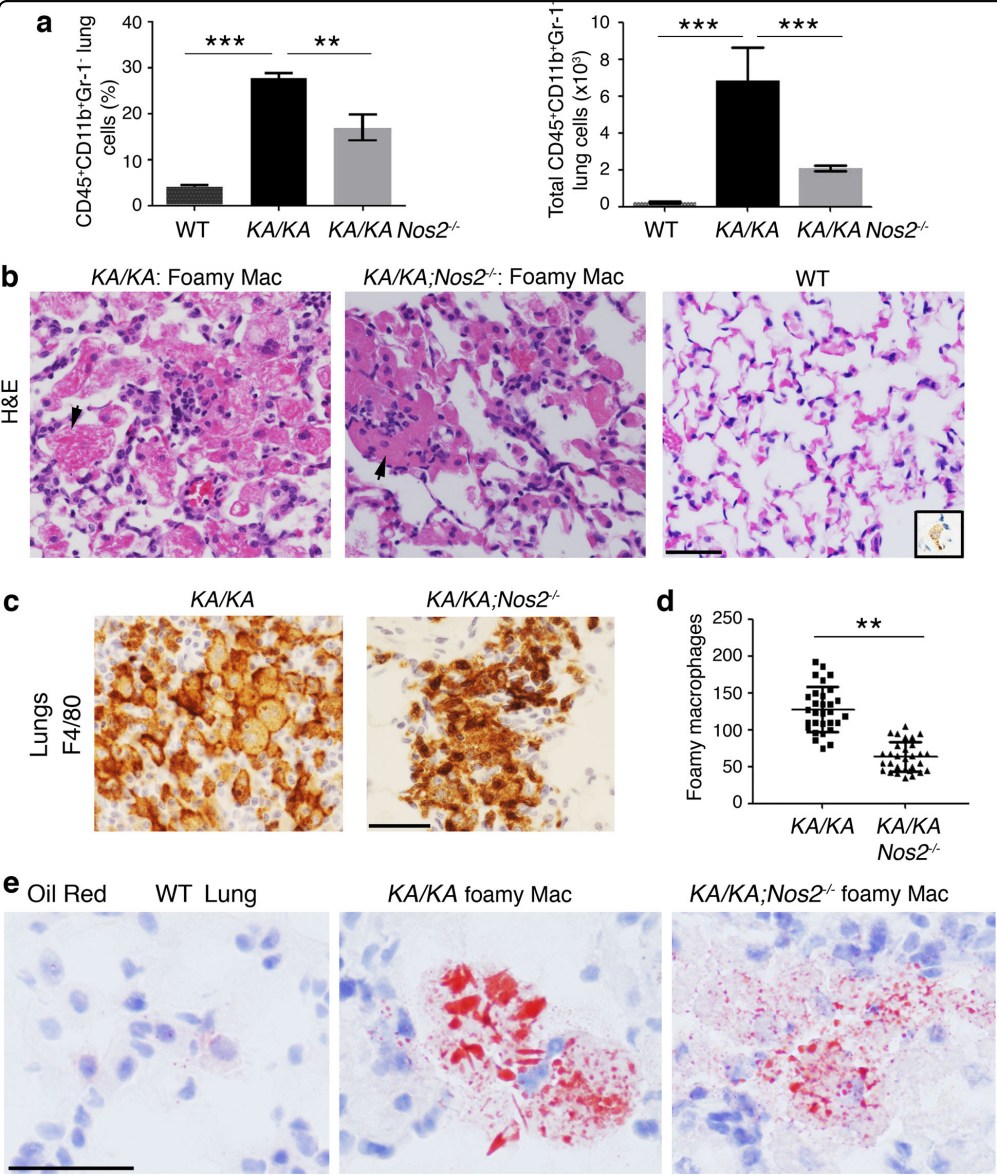
NOS2 deletion reduces infiltrating macrophage and foamy macrophage numbers in *KA/KA* lungs. **a** Flow cytometric analysis compares macrophage (CD45^+^CD11b^+^Gr-1^−^) numbers (% or total) in lungs of WT, *KA/KA*, and *KA/KA;Nos2*^*−/−*^ mice at 5 months of age. Data are statistically analyzed by Student’s *t*-test. Data represent mean ± SD (*n* = 3/group). **p < 0.01; ***p < 0.001. **b** H&E staining for lungs of WT, *KA/KA*, and *KA/KA;Nos2*^*−/−*^ mice. Arrows denote foamy macrophages (Mac). A box at right bottom of WT lung shows a normal macrophage with F4/80-IHC-stained brown color. Scale bar 40 μm. **c** IHC-stained F4/80 Mac (brown color) in lungs of *KA/KA* and *KA/KA;Nos2*^*−/−*^ mice at 4 months of age. Scale bar, 40 μm.** d** Comparison of large foamy Mac, stained by H&E, in lungs of *KA/KA* and *KA/KA;Nos2*^*−/−*^ mice. Data are statistically analyzed by Student’s *t*-test. Data represent mean ± SD (*n* = 3/group). **p < 0.01. **e** Oil red staining for lungs of WT, *KA/KA*, and *KA/KA;Nos2*^*−/−*^ mice. Blue color, nuclear counter staining. Scale bar, 10 μm

Flow cytometric analysis revealed that *KA/KA* lungs had markedly increased numbers of CD45^+^CD11b^+^Gr-1^−^ macrophages compared to WT and that NOS2 deletion significantly reduced macrophage numbers in *KA/KA;Nos2*^*−/−*^ lungs (Fig. 3a). We observed many large foamy macrophages that are characterized by a pink color and needle-shaped crystalline bodies in the cytoplasm due to lipid protein accumulation in *KA/KA* lungs but not in WT lungs, as examined by hematoxylin and eosin (H&E) histological examination (Fig. 3b). A *KA/KA* foamy macrophage can be ten times bigger than a WT macrophage and these foamy cells often form clusters, which indeed expressed F4/80, a macrophage maker (Fig. 3c). NOS2 deletion reduced foamy macrophage numbers and sizes in *KA/KA;Nos2*^*−/−*^ lungs compared to *KA/KA* (Fig. 3c, d). Oil red staining further verified the lipid accumulation in these foamy macrophages in *KA/KA* lungs (Fig. 3e). NOS2 deletion attenuated the intensity of oil red staining in the macrophages and reduced the foamy macrophage numbers, suggesting that NOS2-mediated changes modulate lipid metabolism in the macrophages. Furthermore, we examined expression of PD-L1—an antitumor blockage using a flow cytometry analysis, and found no significant alternations in PD-L1 expression in WT, *KA/KA*, and *KA/KA;Nos2*^*−/−*^ macrophages (Supplementary Figure S3).

### NOS2 deletion does not alter arginase 1 expression but decreases Ym-1 expression in macrophages and NOS2 promotes macrophage migration and survival

**Fig. 4:**
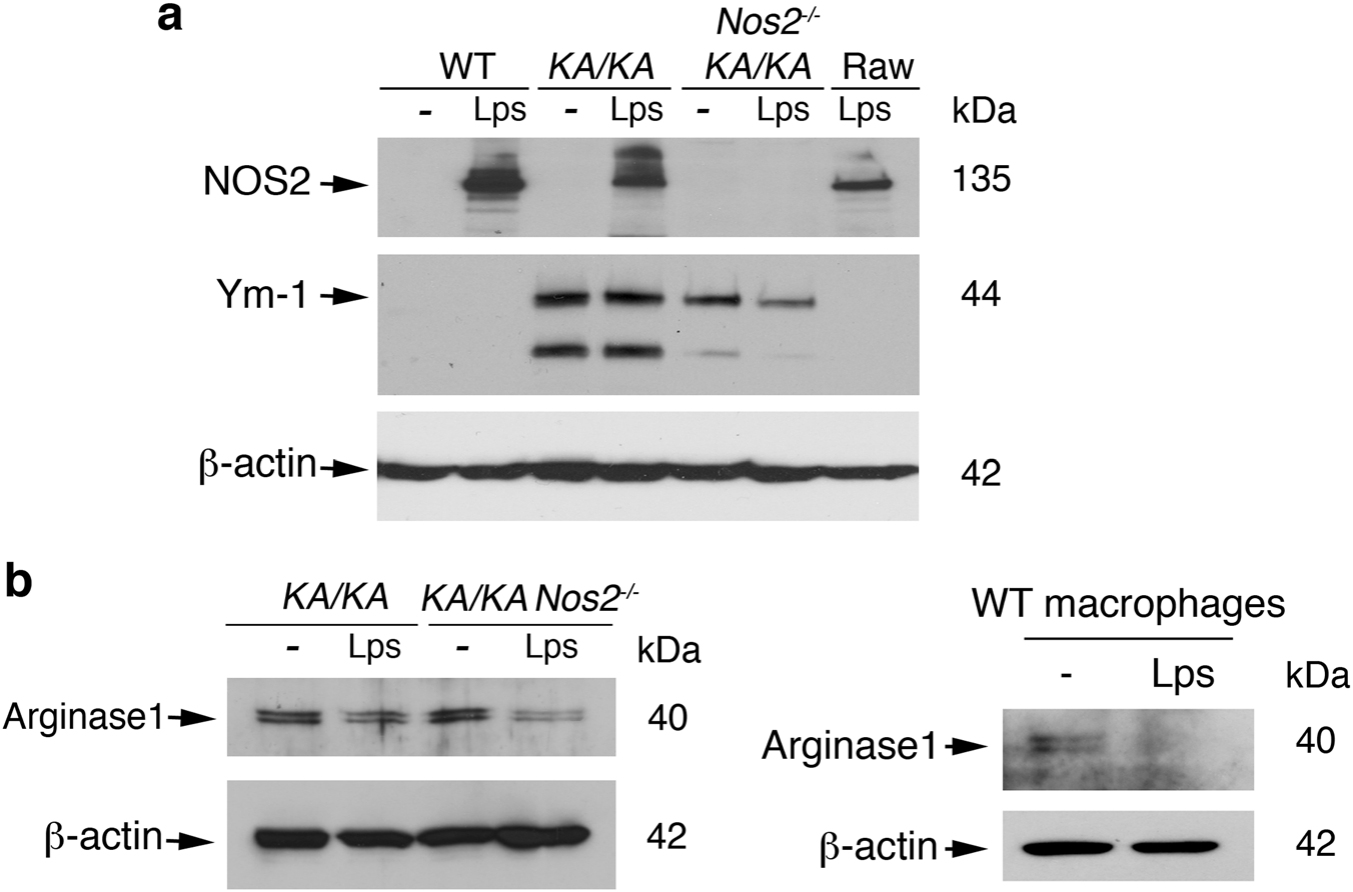
NOS2 affects Ym-1 and arginase 1 expression in macrophages. **a** Western blotting shows NOS2, Ym-1, and arginase 1 levels in WT, *KA/KA*, and *KA/KA;Nos2*^*−/−*^ peritoneal macrophages treated with or without LPS (Lps, 100 ng/ml). Raw, macrophage cell line; β-actin, protein loading control. **b** Western blotting shows arginase 1 levels in WT, *KA/KA*, and *KA/KA;Nos2*^*−/−*^ peritoneal macrophages treated with or without LPS (Lps, 100 ng/ml). β-actin, protein loading control

Previously, we observed increased NOS2 expression in *KA/KA* lung macrophages compared to WT^3^. It is still not clear whether *KA/KA* macrophages expression increased NOS2 or increased inflammation induces NOS2 expression. Thus, we compared induced NOS2 levels in WT, *KA/KA*, and *KA/KA;Nos2*^*−/−*^ peritoneal macrophages following LPS treatment. Raw macrophage cell line was used as a positive control and *KA/KA;Nos2*^*−/−*^ cells were used as a negative control (Fig. 4a). Western blot analysis showed that treatment with LSP induced a comparable level of NOS2 in WT and *KA/KA* macrophages but did not detect NOS2 expression in untreated WT, *KA/KA*, and *KA/KA;Nos2*^*−/−*^ cells (Fig. 4a), suggesting that increased inflammation induces NOS2 expression in *KA/KA* lungs. *KA/KA* macrophages expressed Ym-1 (Fig. 4a), an M2 macrophage marker^9^. NOS2 deletion markedly reduced Ym-1 expression in *KA/KA* macrophages. Treatment with LPS decreased Ym-1 expression in *KA/KA;Nos2*^*−/−*^ macrophages (Fig. 4a). Furthermore, WT lung macrophages expressed lower levels of arginase 1 compared to *KA/KA* macrophages in mice (Supplementary Figure S4). *KA/KA* and *KA/KA;Nos2*^*−/−*^ macrophages expressed comparable levels of arginase 1, another M2 macrophage marker^9^, and treatment with LPS decreased expression of arginase 1 in *KA/KA;Nos2*^*−/−*^ and WT macrophages (Fig. 4b, left and right panels). Of note, the effect of LPS treatment on reduced Ym-1 and arginase 1 expression was more in *KA/KA;Nos2*^*−/−*^ macrophages than in *KA/KA* macrophages, suggesting that NOS2 deletion may reduce the macrophage potential in response to inflammation. These results also indicate that *KA/KA* macrophages were able to express both M1 and M2 markers.

**Fig. 5:**
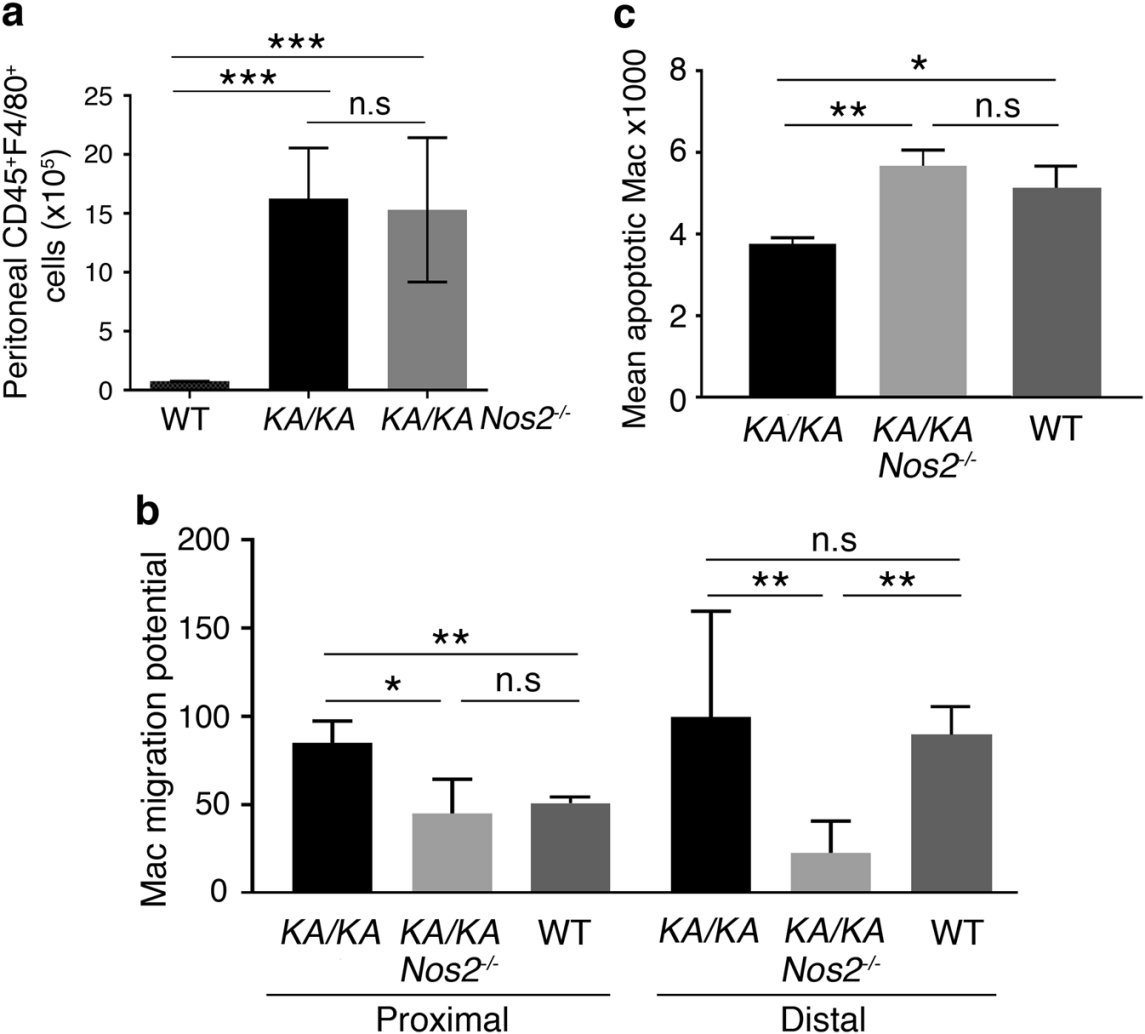
NOS2 regulates macrophage migration and survival. **a** Flow cytometry compares peritoneal macrophage numbers from WT, *KA/KA*, and *KA/KA;Nos2*^*−/−*^ mice at 5 months of age. Data are statistically analyzed by Student’s *t*-test. Data represent mean ± SD (*n* = 3/group). ***p < 0.001; NS, not significant. **b** Macrophage cell migration analysis for WT, *KA/KA*, and *KA/KA;Nos2*^*−/−*^ macrophages at 48 h. The distances of cell migration (see Supplemental Fig. 4B) are statistically analyzed by Student’s *t*-test. Data represent mean ± SD (*n* = 3/group). **p < 0.01; NS, not significant. **c** Flow cytometry analyzes cell apoptosis with a TUNEL kit for *KA/KA* and *KA/KA*;*Nos2*^*−/−*^ macrophages. Data are analyzed by Student’s *t*-test and represent mean ± SD (*n* = 3/group). **p < 0.01

Interestingly, flow cytometric analysis showed similar peritoneal CD45^+^CD11b^+^Gr-1^−^ macrophage numbers in *KA/KA* and *KA/KA;Nos2*^*−/−*^ mice (Fig. 5a) although macrophage numbers were reduced in *KA/KA;Nos2*^*−/−*^ lungs compared to *KA/KA* lungs (Fig. 3a), suggesting that NOS2 deletion may affect the migration of *KA/KA* macrophages. Then, we examined cell migration and found that *KA/KA* macrophages showed a stronger potential of migration for proximal and distal movement than *KA/KA*;*Nos2*^*−/−*^ macrophages did (Fig. 5b; Supplementary Figure S5a). Moreover, *KA/KA* macrophages survived better than *KA/KA*;*Nos2*^*−/−*^ macrophages following TNFα treatment (Fig. 5c; Supplementary Figure S5b). These results suggest that NOS2 induction enhances migration and survival abilities of macrophages in *KA/KA* lungs associated with a sustained chronic inflammation.

### NOS2 null bone marrow (BM) transplantation reduces lung SCC incidence in irradiated *KA/KA* chimeric mice associated with reduced pulmonary macrophages and foamy macrophages

**Fig. 6:**
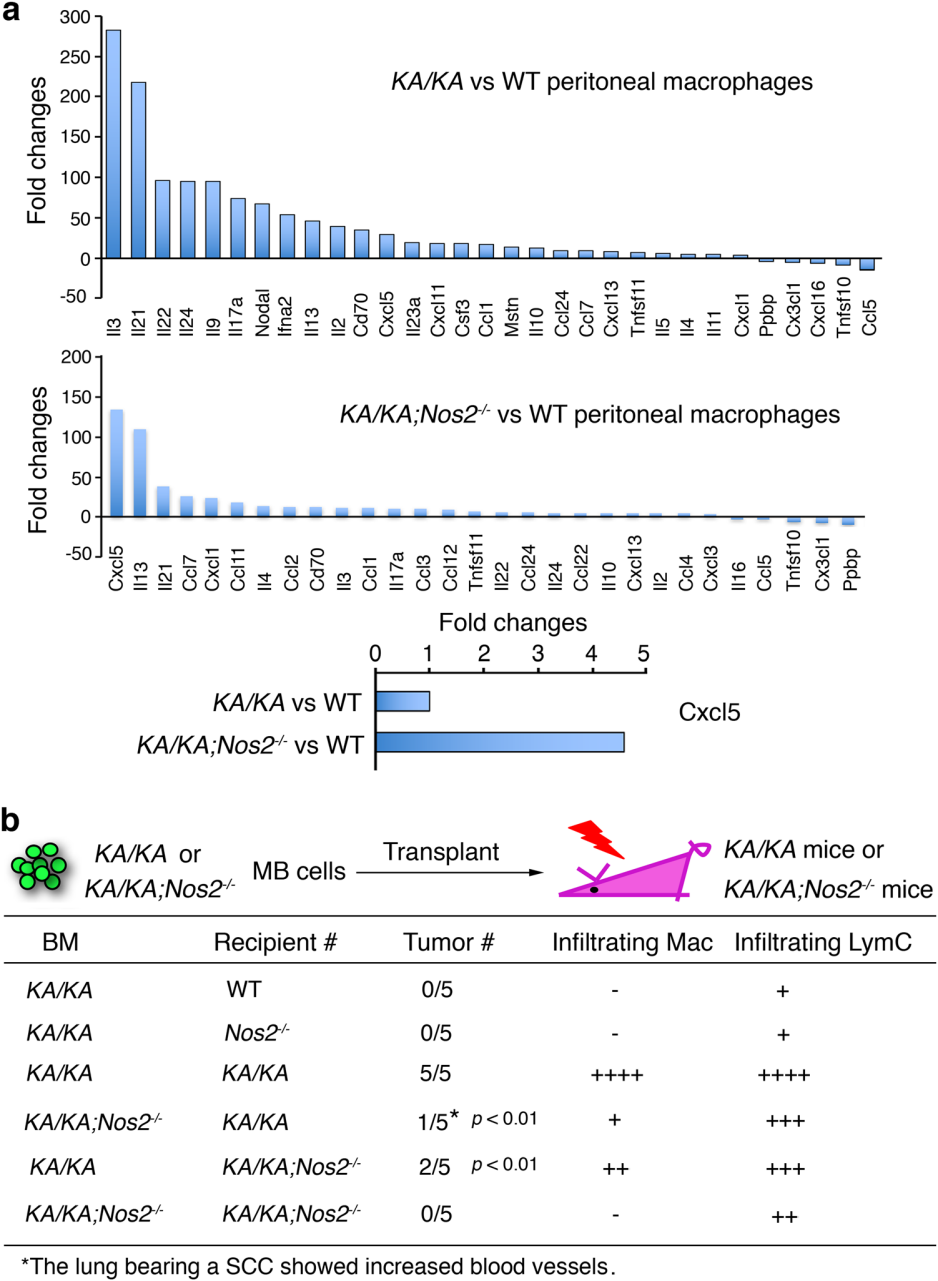
NOS2 regulates macrophage properties and NOS2 null bone marrow (BM) transplantation reduces lung SCC development. **a** Comparison of the expression of multiple cytokines in *KA/KA* vs. WT macrophages or *KA/KA*;*Nos2*^*−/−*^ vs. WT macrophages, as detected by RT^2^ ^2^ Profile PCR Array. Each group contains three samples. **b** Top panel: a scheme for BM transplant experiment. *KA/KA* or *KA/KA;Nos2*^*−/−*^ BM was injected to irradiated *KA/KA* or *KA/KA;Nos2*^*−/−*^ mice. Bottom panel: lung SCC incidence in different irradiated chimeric mice. Infiltrating macrophages (Mac) and lymphocytes (LymC) in the lungs of irradiated *KA/KA* mice receiving *KA/KA* BM were used as positive controls (++++). Infiltrating Mac and LymC in the lungs of irradiated WT mice receiving *KA/KA* BM were used as negative controls (−). Tumor incidence in irradiated *KA/KA* mice receiving *KA/KA;Nos2*^*−/−*^ BM and in irradiated *KA/KA;Nos2*^*−/−*^ mice receiving KA/KA BM was compared with the positive control. Data are analyzed by chi-square test

To understand the effect of NOS2 on inflammatory responses in macrophages, we analyzed expression of multiple cytokines and chemokines in *KA/KA* and *KA/KA*;*Nos2*^*−/−*^ macrophages (Fig. 6a). The analysis showed increased expression of multiple cytokines in *KA/KA* compared to *KA/KA;Nos2*^*−/−*^ peritoneal macrophages (Fig. 6a). IL-3, IL-24, and Nodal promote leukocyte proliferation and differentiation^14–16^. IL-3 promotes the differentiation of myeloid cells to macrophages and IL-17a promotes NOS2 induction^17^. Cxcl5 has been implicated in the limitation of infiltrating foamy macrophages in the plaque area of atherosclerosis^18^. Of note, *KA/KA;Nos2*^*−/−*^ macrophages expressed a marked increase in Cxcl5 compared to *KA/KA* macrophages (Fig. 6a, bottom panel), suggesting that NOS2 deletion-mediated Cxcl5 induction may contribute to the reduction of foamy macrophage numbers.

To determine a physiological effect of macrophage NOS2 deletion on lung SCC development, we performed BM transplant experiments by injecting *KA/KA* BM cells into irradiated *KA/KA* mice or *KA/KA;Nos2*^*−/−*^ mice, or by injecting *KA/KA;Nos2*^*−/−*^ BM cells into irradiated *KA/KA* mice or *KA/KA;Nos2*^*−/−*^ mice (Fig. 6b, top). *KA/KA* BM-induced lung SCCs in all five irradiated *KA/KA* mice, which were used as a positive control for SCC incidence and pulmonary infiltrating leukocytes. *KA/KA* BM-induced lung SCC in two out of five irradiated *KA/KA;Nos2*^*−/−*^ mice (Fig. 6b; Supplementary Figure S6), suggesting that epithelial cell NOS2 also contributes to lung SCC development. *KA/KA;Nos2*^*−/−*^ BM-induced lung SCC in one out of five irradiated *KA/KA* mice and in zero out of five irradiated *KA/KA;Nos2*^*−/−*^ mice (Fig. 6b), suggesting that macrophage NOS2 is required for lung SCC development. WT mice receiving *KA/KA* BM cells and *KA/KA;Nos2*^*−/−*^ mice receiving *KA/KA;Nos2*^*−/−*^ BM cells did not develop any lung SCCs and their lungs did not have increased pulmonary infiltrating inflammatory cells, which were used as negative controls (Fig. 6b). These results confirmed that NOS2 reduces macrophage migration in vivo. In addition, NOS2 also plays a role in the lung epithelial cells during lung SCC development although its mechanism remains to be determined in the future.

## Discussion

In this study, we demonstrate that macrophage NOS2 promotes lung SCC development by circulating inflammatory signaling, enhancing macrophage migration and survival, and impairing lipid metabolism. Our results also explain that an increased inflammatory microenvironment can induce NOS2 expression in *KA/KA* lungs; in turn, NOS2 circulates inflammatory signals between macrophages and epithelial cells, promoting carcinogenesis.

Although we demonstrated that macrophage NOS2 promoted lung SCC development in *KA/KA* mice, BM transplant experiments showed that NOS2 deletion in the epithelial cells reduced lung tumorigenesis associated with decreased infiltrating macrophage numbers in the lungs of irradiated *KA/KA;Nos2*^*−/−*^ mice receiving *KA/KA* BM, compared to irradiated *KA/KA* mice receiving *KA/KA* BM, suggesting that epithelial cell NOS2 is required for circulating inflammation. Furthermore, Ki67-stained proliferating basal cells of the lung bronchi in *KA/KA;Nos2*^*−/−*^ mice were significantly decreased compared to *KA/KA* lungs, suggesting that NOS2 deletion reduces the basal cell growth in the lung bronchi or that NOS2 promotes epithelial cell growth in *KA/KA* lungs through a loop of inflammatory cells and epithelial cells.

In this study and another^3^, we showed that lung tumor associated macrophages (TAMs) expressed increased NOS2, arginase 1, and Ym-1 in *KA/KA* mice, suggesting that *KA/KA* TAMs possess both M1 and M2 features. NOS2 deletion decreased the expression of Ym-1 in *KA/KA;Nos2*^*−/−*^ macrophages compared to *KA/KA* macrophages and LPS treatment in vitro further reduced Ym-1 and arginase 1 expression in *KA/KA;Nos2*^*−/−*^ macrophages, indicating that NOS2 deletion attenuates the M2 potential of *KA/KA* macrophages, as well as reduces responses to inflammatory signaling in macrophages in vivo.

In addition to constitutively expressed NOS-1 and NOS-3 in vivo, NOS activity has been reported in several bacterial species (bNOS), including *Staphylococcus aureus* and the notorious pathogens *Bacillus anthracis*^19^. *S. aureus* is a member of the normal flora of the body and is frequently found in the nose, in the respiratory tract, and on the skin. Thus, these NOS isoforms may provide NO required for maintaining NO’s essential functions in the absence of NOS2.

The BM-derived macrophages can differentiate to foamy macrophages^20^ and the foamy macrophages associated with impaired lipid metabolism can drive inflammation^8,21^; however, the relationship between foamy macrophages and lung tumor development has not be investigated. In this study, we described large foamy macrophages associated with accumulated lipid proteins in *KA/KA* lungs, which was associated with lung SCC development. NOS2 deletion reduced foamy macrophage numbers and sizes and NOS2 null BM transplantation reduced lung tumorigenesis with decreased pulmonary macrophage infiltration, suggesting that increased NOS2 may impair the lipid metabolism in macrophages during lung carcinogenesis. Cxcl5 eliminates foamy macrophages^18^. NOS2 deletion increased Cxcl5 expression in *KA/KA* macrophages, suggesting that NOS2’s effect on the macrophage property may be associated with Cxcl5 regulation. The detailed mechanism underlying the lipid metabolism in macrophages regulated by NOS2 remains to be investigated in the future. Also, we will investigate whether lung SCC induces foamy macrophages in the future. The increased inflammasome activity has been reported in *KA/KA* macrophages^22^, which may contribute to the increased expression of cytokines, chemokines, and NOS2. Overall, this finding identifies a new function of macrophage NOS2 in lung carcinogenesis, as well as provides new insight into therapeutic strategies for prevention and treatment of lung SCC.

## Materials and methods

### Mice

The animal procedures (14-051 and 14-052) for all animal experiments were reviewed and approved by the Animal Care and Use Committee of the National Cancer Institute. *L-Ikkα*^*KA/KA*^ (*KA/KA*) mice express a kinase-dead IKKα with an amino acid K44 > A44 substitution, and also specifically express a WT IKKα in the skin under the loricrin promoter control for protecting the skin integrity, which was previously described^3,23^. *Nos2*^*−/−*^ mice were purchased from Jackson laboratory (Cat# 002609). All the mice used for this study have been crossed over nine generations to FVB background.

### Antibodies

The following antibodies were used for western blotting and immunostaining, including COX2 (sc-1747-R), NOS2 (sc-651), and arginase 1 (sc-20150, H-52) from Santa Cruz Biotechnology, Inc., Santa Cruz, CA; TRIM29 (ab22207); Chitinase like 3 protein 3 (Ym-1, ab93034), and 8-OHdG (ab26842) from Abcam, CHEMICON International, Inc., Temecula, CA; and β-actin (A2228) from Sigma-Aldrich.

### Histopathology, western blot analysis, and immunohistochemical (IHC) and immunofluorescence staining

The Histology and Tissue Core Facility at the Frederick National Laboratory for Cancer Research routinely prepared paraffin sections of mouse organs and performed hematoxylin and eosin (H&E) staining and IHC staining for K5, CD3, and F4/80. Eight micrometers frozen sections was immunofluorescently stained overnight at 4 °C with anti-8-OHdG antibody, and were then washed and stained with the fluorescence-conjugated secondary antibodies for 2 h at room temperature. Finally, the sections were mounted with diamidino-2-phenylindole (Vector Laboratories, Burlingame, CA) mounting medium^3^. Cell lysates (15 μg) or protein extract from the tissues (20 μg) were examined in acrylamide gels using western blotting with specific antibodies and were visualized by chemiluminescence, as previously described^24^. Masson’s trichrome stain was performed by Histoserve, Inc.

### Array analysis for gene expression profiles

Total RNA was isolated from the lungs of 14-week-old mice (WT, *KA/KA*, and *KA/KA*;*Nos2*^*−/−*^ mice) using the kit, according to TRIzol (Invitrogen) manufacturer instructions and given to the Center for Cancer Research Genomics Laboratory to perform GeneChip Mouse Genome Array 430 2.0. Raw data were then analyzed using Partek Genomics Suite 2.0 software. The statistical significance of expression of interested genes was sorted or grouped. Microarray results (accession number: GSE101759) have been deposited at the National Center for Biotechnology Information Gene Expression Omnibus (http://www.ncbi.nlm.nih.gov/geo/).

### Cell isolation and flow cytometry

For pulmonary leukocyte isolation, lungs were cut into small pieces and then digested in RPMI medium containing 5% fetal calf serum, 250 U/ml of type IV collagenase (Invitrogen), 100 mg/ml of DNase I (Roche), and 1 mmol/l of ethylenediaminetetraacetic acid (pH 8.0), at 37 °C for 30 min. The homogenate was then filtered with a 70-mm nylon cell strainer (BD Biosciences), washed with HBSS medium, and resuspended in 40% Percoll (Amersham Pharmacia) in Dulbecco’s modified Eagle’s medium (BioWhittaker). The suspension was underlaid with 80% Percoll and centrifuged for 25 min at 1000×*g*. Leukocytes were collected from the interphase, washed, and enumerated on Sysmex KX-21. Fluorescent-conjugated antibodies used for analyses included CD3 (clone 500A2, 145-2C11), CD8 (clone 53–6.7), and CD4 (clone GK1.5), which were obtained from BD Biosciences Pharmingen; MHC II (clone M5/114.15.2), Gr-1 (clone RB6-8C5), B220 (clone RA3-6B2), CD11c (clone N418), F4/80 (clone BM8), CD11b (clone M1/70), and PD-L1 (12-5983-42), which were obtained from eBioscience; and CD45 (clone 30-F11), which was obtained from Invitrogen. Labeled cells were analyzed on an LSR II flow cytometer (Becton Dickinson). Data were analyzed with FCS Express software.

### Isolation of mouse peritoneal macrophages

Ten-week-old female or male WT, *KA/KA*, and *KA/KA;Nos2*^*−/−*^ mice were killed and 70% ethanol was immediately used for disinfection of animal fur. Pulling the abdominal skin with forceps, making a small incision over the caudal half of the abdomen with scissors, and exposing the underlying abdominal wall by retraction were performed; care was taken pierce the abdominal wall while incising the skin. Following lifting the abdominal wall with forceps, 10 ml sterile PBS was injected into the caudal half of the peritoneal cavity using a 25-gauge needle. The entire mouse body was gently shaken for 30 s. Then, PBS containing resident peritoneal cells was slowly withdrawn by inserting a 25-gauge needle into the cranial half of the peritoneal cavity. Cells were collected by centrifuging at 1500×*g* for 8 min, and the pellet was resuspended with DMEM medium with 10% FBS and 1% penicillin–streptomycin-l–glutamine for cell counting. Cells were seeded 1 × 10^5^/ml and replaced with fresh RPMI medium supplemented with 10% FBS 2–3 h later. For the experimental group, isolated macrophages were stimulated with 100 ng/ml LPS for 18 or 12 h, and then total RNA or protein was collected.

### Reverse transcription polymerase chain reaction (RT-PCR)

Total RNA was isolated from the lung tissues using the TRI Reagent (Molecular Research Center, Inc., Cincinnati, OH). Complementary DNA was synthesized with a SuperScript®III First-Strand kit (Invitrogen, Cat no. 18080-051, CA). PCR conditions were used: one cycle at 95 °C for 4 min; 35 cycles at 95 °C for 30 s, 58 °C for 30 s, and 72 °C for 50 s; and one cycle at 72 °C for 7 min.

### Quantitative real-time PCR

Total RNA was isolated from lung homogenates using TRIzol (Invitrogen) and was precipitated and reverse transcribed (Applied Biosystems). The Mouse Inflammatory Cytokines and Receptors RT^2 ^Profiler PCR Array (PAMM-011E, SuperArray), obtained from SABiosciences (Frederick, MD), was used to detect the expression of murine cytokines and chemokines, according to the manufacturer’s directions. Genes of interest were subsequently examined using real-time RT-PCR with the TaqMan Universal PCR Master Mix and the ABI Prism 7300 Detection System (TaqMan; Applied Biosystems) according to the manufacturer’s instructions. Gene expression was normalized to the level of the β-actin housekeeping gene. Data were expressed as a fold change in mRNA expression relative to control values.

### BM transfer

Recipient 4- or 5-week-old WT, *KA/KA*, and *KA/KA;Nos2*^*−/−*^ mice on an FVB background were irradiated with 950 rad using the 137Cs irradiator facility at the Frederick National Laboratory for Cancer Research. The irradiated mice were intravenously injected with 1 × 10^6^ BM cells isolated from 10-week-old *KA/KA* or *KA/KA;Nos2*^*−/−*^ mice^25^. Single-cell suspensions were prepared by passing the dissociated cells from the femur and tibia through a 40-μm cell strainer and then washing with PBS.

### Wound-healing assay for macrophage migration

Isolated macrophages (1.5 × 10^6^/ml) were plated onto coated cell culture plates with two-well inserts (Ibidi) in DMEM with 10% FBS and 1% penicillin–streptomycin. Cell suspension was added to the wells, allowing for adherence and growth in designated areas only. After 24 h, the insert was removed and then the adherent monolayer of cells was washed with PBS, photographed in phase contrast with a Nikon microscope (*T* = 0 h), and placed back into complete growth medium with or without treatment with LPS (200 ng/ml). The cells were photographed once following 48 h of incubation at 37 °C (*T* = 48 h). The distance between the two groups of cells was measured at 0 h using Nikon imaging software and superimposed on the image of cells at 48 h. Cells within the boundary were then counted and sorted by distance migrated (e.g., proximal = < 1/10^th^ original distance, distal = > 1/10^th^ original distance).

### Apoptosis assay

Peritoneal cells collected from WT, *KA/KA*, and *KA/KA;Nos2*^*−/−*^ mice were seeded in six-well culture dishes and washed with PBS following adherence. Remaining peritoneal macrophages were then stimulated with TNFα (10 ng/ml) for 72 h. Following treatment, cells were collected and prepared, according to APO-BrdU TUNEL Assay Kit (Invitrogen, Cat# A23210) instructions and detected using flow cytometry. The percent and mean number of apoptotic cells were recorded as anti-BrdU positive events (per 2.5 × 10^5^ cells). The significance of the data was determined by statistical analysis and Student’s *t*-test.

### Statistical analysis

Statistical analysis methods were indicated in the figure legends. *P*-values <0.05 were considered statistically significant.

## Electronic supplementary material

CDDis Supplementary Figures(PDF 6652 kb)
