# Supplementary material for: Macrophage inducible nitric oxide synthase circulates inflammation and promotes lung carcinogenesis
Source: Cell Death Discov. 2018 Mar 26;4:46. doi: 10.1038/s41420-018-0046-5 (PMC5967330; doi:10.1038/s41420-018-0046-5)
Supplement: Supplementary file 1 — CDDis Supplementary Figures(PDF 6652 kb) [file 41420_2018_46_MOESM1_ESM.pdf]

Supplementary Data

Supplementary Figure 1

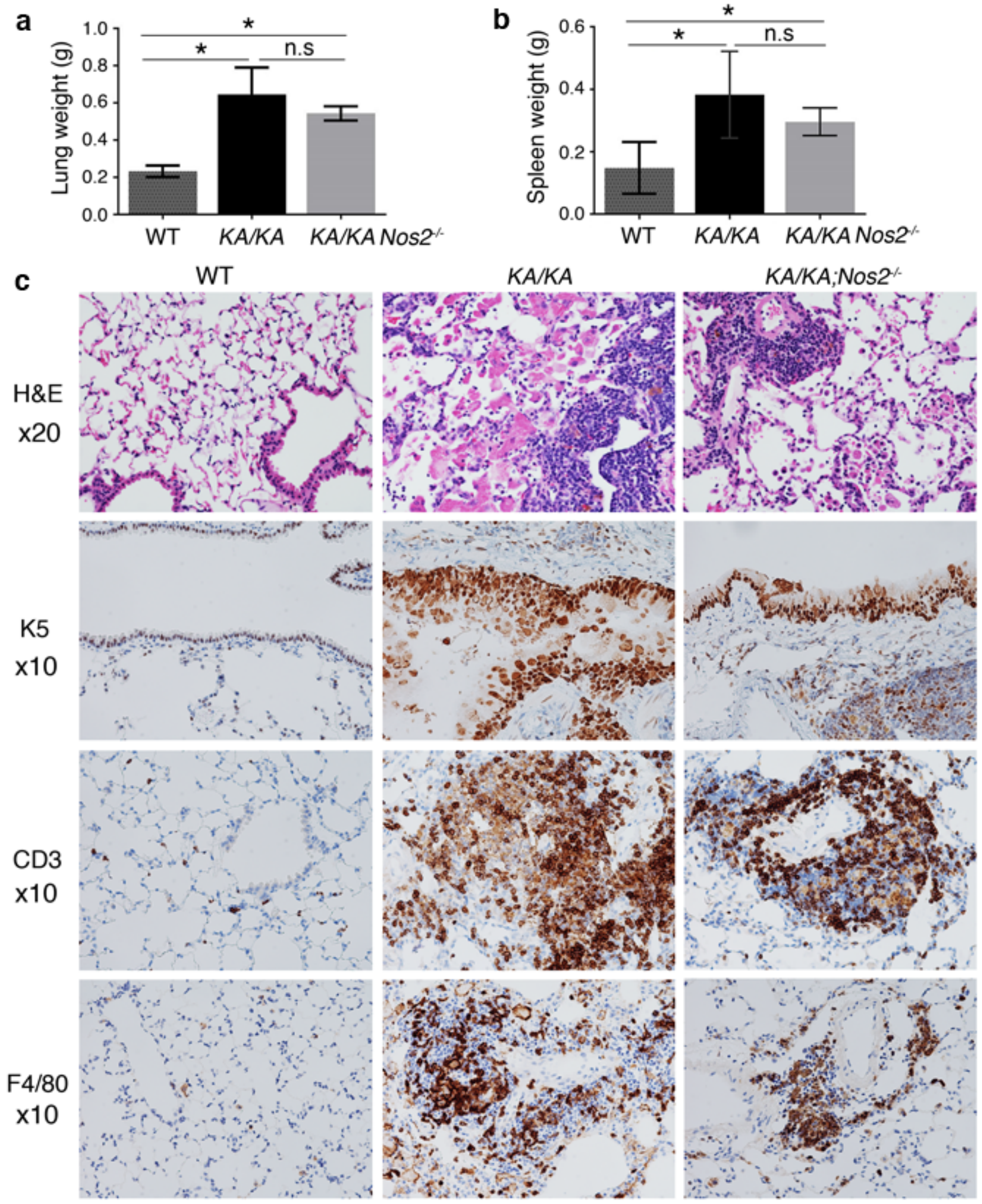

Supplementary Figure 1. H&E and IHC staining for *KA/KA* and *KA/KA;Nos2*$^{-/-}$ lungs.
(**a**–**b**) Comparing lung and spleen weights of WT, *KA/KA*, and *KA/KA;Nos2*$^{-/-}$ mice at 4 months of age. Data are statistically analyzed by Student's t-test and represent mean ± SD (n = 3/group). *, $p < 0.05$; n.s, no statistical significance.
(**c**) H&E staining, immunohistochemistry (IHC) for K5, CD3, and F4/80 examine the lungs of WT, *KA/KA*, and *KA/KA;Nos2*$^{-/-}$ mice. Brown color, positive. Original magnification × 200 (x20) and × 100 (x10).

Supplementary Figure 2

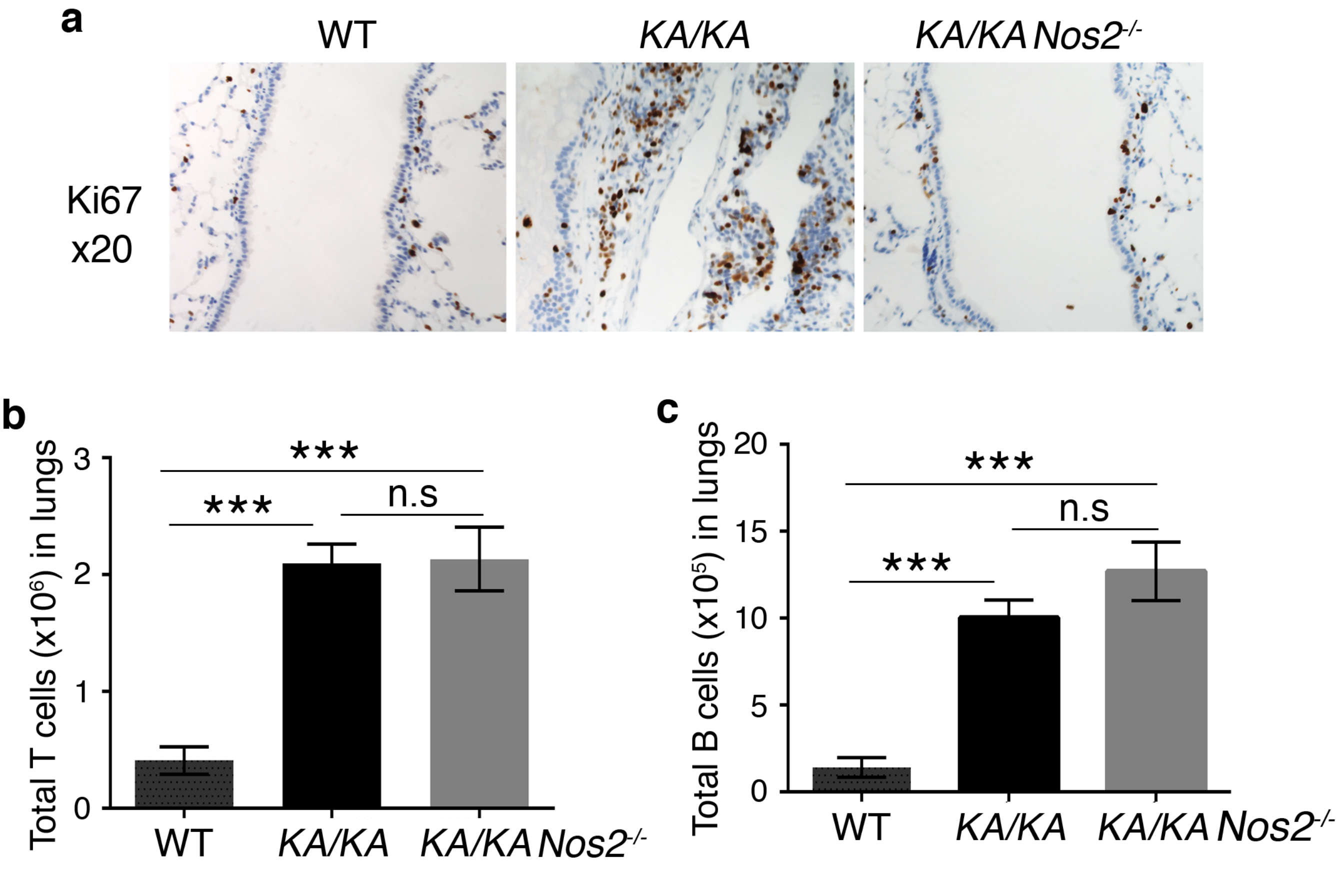

Supplementary Figure 2. The effect of NOS2 deletion on lymphocyte numbers.
(**a**) Ki67 IHC staining for the basal cells of lung bronchi in WT, *KA/KA*, and *KA/KA;Nos2*$^{-/-}$ mice. Dark brown, positive staining; blue, nuclear counting staining. Original magnification × 200 (x20).
(**b**–**c**) Numbers of T cells and B cells in the lungs of WT, *KA/KA*, and *KA/KA;Nos2*$^{-/-}$ mice, examined by flow cytometry and statistically analyzed by Student's t-test. Data represent mean ± SD (n = 3/group). ***, $p < 0.001$; n.s, not significant.

Supplementary Figure 3

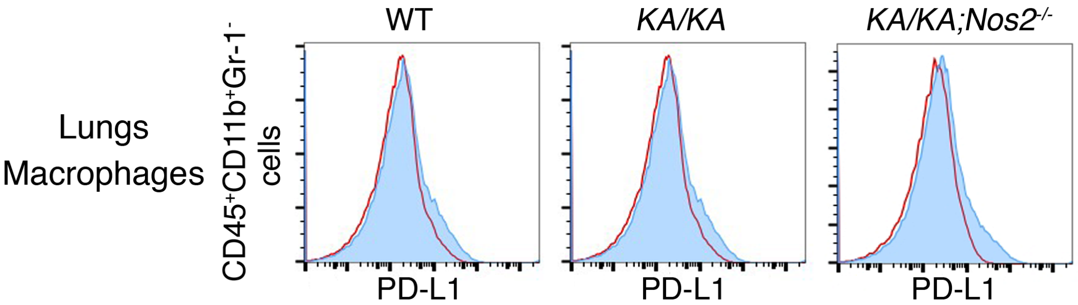

Supplementary Figure 3. Effects of NOS2 deletion on PD-L1 of macrophages.
Flow cytometric analysis shows PD-L1 levels in macrophages from the lungs of WT, *KA/KA*, and *KA/KA;Nos2$^{-/-}$* mice at 4 months of age.

Supplementary Figure 4

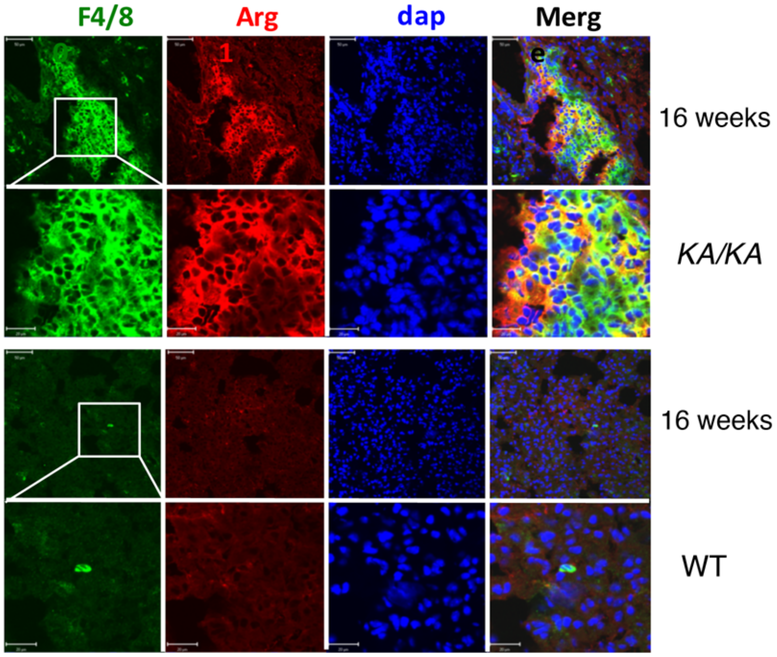

Supplementary Figure S4. Analysis for macrophages and immunofluorescencent staining for lung macrophages of *KA/KA* and WT mice at 10 weeks of age.
IF staining for macrophages with anti-F4/80 and anti-arginase 1 (Arg) antibodies. dap, blue, DAPI. Scale bar, 50 μm.

Supplementary Figure 5

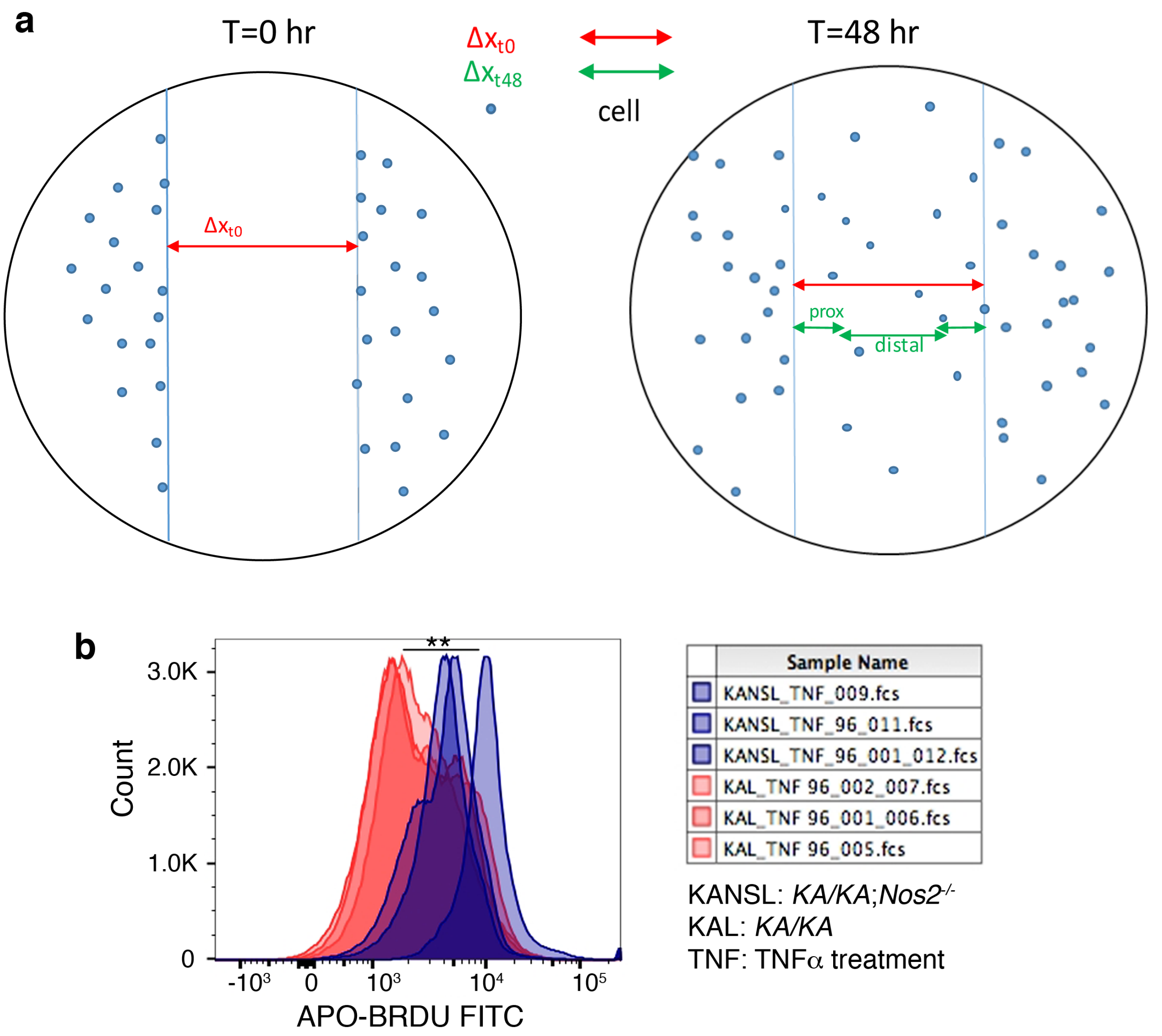

Supplementary Figure 5. Effect of NOS2 on macrophage migration and survival.
(**a**) The method used for measuring macrophage migration after plating cells treated with LPS at 48 hr. Two groups include proximate (pro) and distal migration of cells (see Fig. 5B). The detailed method was described in Methods.
(**b**) The profile of flow cytometric analysis for cell apoptosis after TNFα treatment (see Fig. 5C).

Supplementary Figure 6

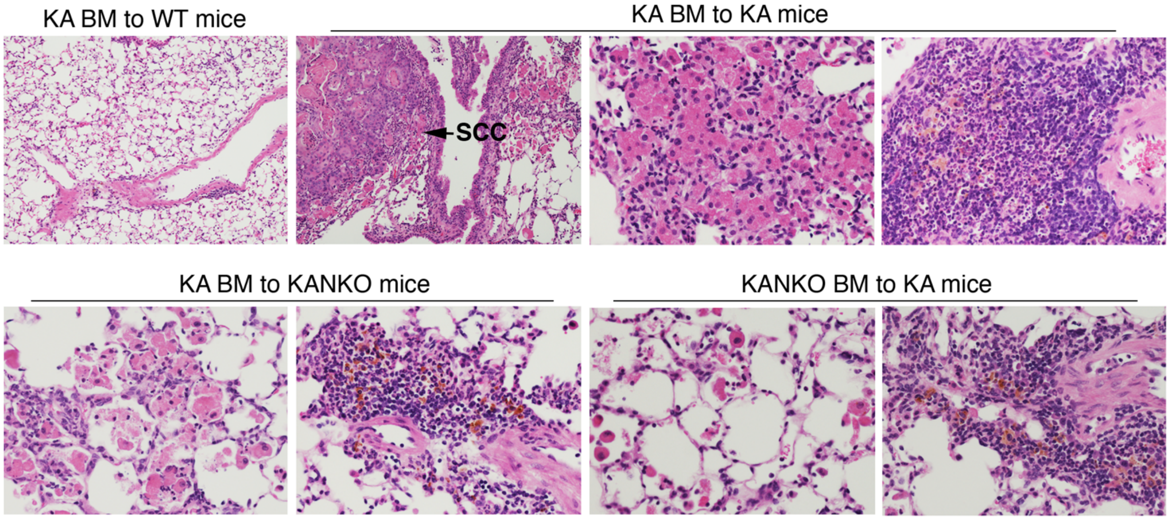

Supplementary Figure 6. H&E staining shows the lungs of different irradiated chimeric mice. KA, *KA/KA*; BM, bone marrow; KANKO, *KA/KA;Nos2*$^{-/-}$. SCC, squamous cell carcinoma. Original magnification × 4 for first two panels of top left and original magnification × 20 for the rest panels.
